# Homœopathy Condemned by the Law

**Published:** 1850-08

**Authors:** 


HOMŒOPATHY CONDEMNED BY THE LAW.

*Marine Court.—Before Judge Lynch. Homœopathy.*

*E. Rossi Corsi* v. *Max Maretzek.*—To recover $100, balance for services as singer at the Opera House, the defence to which was that plaintiff had incurred a forfeiture of $100 by not performing, or personally giving notice of illness, under certificate of the Doctor of the Opera House, on February 23, agreeable to the regulations already referred to. The decision in this case as to Homœopathic physicians having been somewhat misunderstood, we give the portion of the opinion which refers to it.

"The Court, after stating that it was shown by physician of plaintiff, and who it is said was proved to be a regular M. D., that plaintiff was unable that night to perform, and expressing its concurrence in the view that at such an establishment as the Opera House, strict discipline should be maintained, and the penalties upheld, says : 'But I feel bound to regard the rule of evidence which requires in cases of penalty and forfeiture, strict proof of its being incurred—the objection to the evidence of the attending physician is technical and strict, and before defendant can avail himself of it, he must show that he has fully complied with what was to be done on his part. The rule stuck up at the Opera House is in these words : ' Sickness must be proved by the *doctor* employed by the director.' Now, though it is proven there was a notice posted up in the Opera House that Dr. Quinn was employed by the director, yet *it has not been proved on this trial that Dr. Quinn was a doctor, or that he had taken a degree as Doctor of Medicine, or that he was authorized by the Medical Society, or had a regular license to practice, which I think was necessary in order to constitute him a doctor, and to show a regular appointment under the rule,* and which I do not feel at liberty in such cases to supply by inference. So far as there is evidence on the subject, it went to show that *Dr. Quinn practiced upon principles of homœopathy, and that such practitioners are not recognized by the faculty of medicine, nor by a majority of the public, as regular practitioners.* Under these circumstances, I am of opinion that plaintiff was authorized to make proof of his sickness by his attending physician; and as such proof was made to my satisfaction, I think the plaintiff is not subject to the fine, and give judgment in his favor for $100, the amount.' "

